# Modified cosmology through nonextensive horizon thermodynamics 

**DOI:** 10.1140/epjc/s10052-018-6480-y

**Published:** 2018-12-05

**Authors:** Andreas Lymperis, Emmanuel N. Saridakis

**Affiliations:** 1 Department of Physics, University of Patras, 26500 Patras, Greece; 2 Department of Physics, National Technical University of Athens, Zografou Campus GR, 157 73 Athens, Greece; 3 CASPER, Physics Department, Baylor University, Waco, TX 76798-7310 USA

## Abstract

We construct modified cosmological scenarios through the application of the first law of thermodynamics on the universe horizon, but using the generalized, nonextensive Tsallis entropy instead of the usual Bekenstein–Hawking one. We result to modified cosmological equations that possess the usual ones as a particular limit, but which in the general case contain extra terms that appear for the first time, that constitute an effective dark energy sector quantified by the nonextensive parameter $\delta$. When the matter sector is dust, we extract analytical expressions for the dark energy density and equation-of-state parameters, and we extend these solutions to the case where radiation is present too. We show that the universe exhibits the usual thermal history, with the sequence of matter and dark-energy eras, and according to the value of $\delta$ the dark-energy equation-of-state parameter can be quintessence-like, phantom-like, or experience the phantom-divide crossing during the evolution. Even in the case where the explicit cosmological constant is absent, the scenario at hand can very efficiently mimic $\Lambda \hbox {CDM}$ cosmology, and is in excellent agreement with Supernovae type Ia observational data.

## Introduction

Recent cosmological observations from various and different fields reveal that the universe has experienced two accelerated expansion phases, one at early and one at late times. Since the established knowledge of general relativity and Standard Model of particles is not sufficient to explain this behavior, there has been a lot of effort in constructing theories beyond the above, in order to acquire the necessary extra degrees of freedom. On one hand, one can introduce new forms of matter, such as the inflaton field [1, 2] or the concept of dark energy [3, 4], which in the framework of general relativity can lead to the aforementioned accelerated behaviors. On the other hand, one can construct gravitational modifications, which possess general relativity as a particular limit, but at large scales can provide extra degrees of freedom capable of driving the acceleration (for reviews see [5–8]). Note that this last approach has the additional theoretical advantage that may improve renormalizability, which seems to be necessary towards quantization [9, 10].

The usual approach of constructing modified gravitational theories is to start from the Einstein–Hilbert action and add correction terms. The simplest extension is to replace the Ricci scalar *R* by a function *f*(*R*) [11–14]. Similarly, one can proceed in constructing many other classes of modification, such as *f*(*G*) gravity [15, 16], Lovelock gravity [17, 18], Weyl gravity [19, 20] and Galileon theory [21–23]. Alternatively, one can start from the torsional formulation of gravity and build various extensions, such as *f*(*T*) gravity [24–26], $f(T,T_G)$ gravity [27, 28], etc.

On the other hand, there is a well-known conjecture that one can express the Einstein equations as the first law of thermodynamics [29–31]. In the particular case of cosmology in a universe filled with the matter and dark-energy fluids, one can express the Friedmann equations as the first law of thermodynamics applied in the universe apparent horizon considered as a thermodynamical system [32–35]. Reversely, one can apply the first law of thermodynamics in the universe horizon, and extract the Friedmann equations. Although this procedure is a conjecture and not a proven theorem, it seems to work perfectly in a variety of modified gravities, as long as one uses the modified entropy relation that corresponds to each specific theory [35–44]. Nevertheless, note that in order to know the modified entropy relation of a modified gravity, ones needs to know this modified gravity a priori and investigate it in spherically symmetric backgrounds. In this sense the above procedure cannot provide new gravitational modifications, offering only a way to study their features.

In the present work we are interested in following the above procedure in a reverse way, in order to construct new cosmological modifications. In particular, we will apply the first law of thermodynamics, but instead of the usual entropy relation we will use the nonextensive, Tsallis entropy [45–47], which is the consistent generalization of the Boltzmann–Gibbs additive entropy in non-additive systems, such as gravitational ones. In this way we will obtain new modified Friedmann equations that possess the usual ones as a particular limit, namely when the Tsallis generalized entropy becomes the usual one, but which in the general case contain extra terms that appear for the first time. Hence, we will investigate in detail the cosmological implications of these new extra terms.

The plan of the work in the following: In Sect. 2 we present the construction of the scenario, applying the first law of thermodynamics in the universe horizon, but using the generalized, nonextensive Tsallis entropy instead of the usual Bekenstein–Hawking one. In Sect. 3 we investigate the cosmological evolution, focusing on the behavior of the dark energy density and equation-of-state parameters, studying separately the cases where an explicit cosmological constant is present or absent. Finally, in Sect. 4 we summarize our results.

## The model

In this section we present the scenario at hand, namely we extract modified Friedmann equations applying the first law of thermodynamics to the whole universe, but using the generalized Tsallis entropy instead of the standard one. Throughout the work we consider a homogeneous and isotropic Friedmann–Robertson–Walker (FRW) geometry with metric

1 $$\begin{aligned} ds^2=-dt^2+a^2(t)\left( \frac{dr^2}{1-kr^2}+r^2d\Omega ^2 \right) , \end{aligned}$$

where *a*(*t*) is the scale factor, and with $k=0,+\,1,-\,1$ corresponding to flat, close and open spatial geometry respectively.

### Friedmann equations as the first law of thermodynamics

Let us first briefly review the extraction of the Friedmann equations in the case of general relativity, from the application of the first law of thermodynamics. We start by considering the expanding universe filled with the matter perfect fluid, with energy density $\rho _m$ and pressure $p_m$. Although it is not trivial what it should be its “radius”, namely the length that forms its boundary, there is a consensus that one should use the apparent horizon [32, 33, 48]

2 $$\begin{aligned} \tilde{r}_a=\frac{1}{\sqrt{H^2+\frac{k}{a^2}}}, \end{aligned}$$

with $H=\frac{\dot{a}}{a}$ the Hubble parameter and dots denoting derivatives with respect to *t*. The apparent horizon is a marginally trapped surface with vanishing expansion, defined in general by the expression $h^{ij}\partial _i\tilde{r}\partial _j \tilde{r}=0$ (which implies that the vector $\nabla \tilde{r}$ is null or degenerate on the apparent horizon surface) [49]. For a dynamical spacetime, the apparent horizon is a causal horizon associated with the gravitational entropy and the surface gravity [49–51]. Finally, note that in flat spatial geometry the apparent horizon becomes the Hubble one.

The crucial point in the application of thermodynamics in cosmology is that the first law is interpreted in terms of energy flux and area of local Rindler horizons, and that heat is defined as energy that flows across a causal horizon, and hence thermodynamics is applied on the horizon itself, considered as a system separated not by a diathermic wall but by a causality barrier [29–31]. One can attribute to the universe horizon a temperature and an entropy that arise from the corresponding relations of black hole temperature and entropy respectively, but with the universe horizon, namely the apparent horizon, in place of the black hole horizon. Concerning the black hole temperature, it is well known that for spherically symmetric geometry its relation does not depend on the underlying gravitational theory, and it is just inversely proportional to the black hole horizon, namely $T=1/(2\pi r_h)$ [52]. Hence, one can attribute to the universe horizon the temperature [31]

3 $$\begin{aligned} T_h=\frac{1}{2\pi \tilde{r}_a}, \end{aligned}$$

independently of the gravitational theory that governs the universe. Concerning the back hole entropy, it is also known that its relation does depend on the underlying gravitational theory [31]. In the case of general relativity one obtains the usual Bekenstein–Hawking relation $S=A/(4G)$ (in units where $\hbar =k_B = c = 1$), where $A=4\pi r_h^2$ is the area of the black hole and *G* the gravitational constant. Thus, in the case of a universe governed by general relativity, the horizon entropy will be just

4 $$\begin{aligned} S_h=\frac{1}{4G} A. \end{aligned}$$

Finally, a last reasonable assumption is that after equilibrium establishes the universe fluid acquires the same temperature with the horizon one, otherwise the energy flow would deform this geometry [53].^1^

As the universe evolves an amount of energy from the universe fluid crosses the horizon. During an infinitesimal time interval *dt*, the heat flow that crosses the horizon can be straightforwardly found to be [33]

5 $$\begin{aligned} \delta Q=-dE=A(\rho _m+p_m)H \tilde{r_{a}}dt, \end{aligned}$$

with $A=4\pi r_{a}^2$ the apparent horizon area. On the other hand, the first law of thermodynamics states that $-dE=TdS$. Since the temperature and entropy of the horizon are given by (3) and (4) respectively, we find that $dS=2\pi \dot{\tilde{r}}_a dt/G$, with $\dot{\tilde{r}}_a$ easily obtained from (2). Inserting the above into the first law of thermodynamics we finally acquire

6 $$\begin{aligned} -4\pi G (\rho _m +p_m)= \dot{H} - \frac{k}{a^2}. \end{aligned}$$

Additionally, assuming that the matter fluid satisfies the conservation equation

7 $$\begin{aligned} \dot{\rho }_m +3H(\rho _m +p_m)=0, \end{aligned}$$

inserting it into (6) and integrating we obtain

8 $$\begin{aligned} \frac{8\pi G}{3}\rho _m =H^2+\frac{k}{a^2}-\frac{\Lambda }{3}, \end{aligned}$$

with $\Lambda$ the integration constant, that plays the role of a cosmological constant.

Interestingly enough, we saw that applying the first law of thermodynamics to the whole universe resulted to the extraction of the two Friedmann equations, namely Eqs. (6) and (8). The above procedure can be extended to modified gravity theories too, where as we discussed the only change will be that the entropy relation will not be the general relativity one, namely (4), but the one corresponding to the specific modified gravity at hand [35–44]. Nevertheless, we have to mention here that although the above procedure offers a significant tool to study the features and properties of various modified gravities, it does not lead to new gravitational modifications, since one needs to know the entropy relation, which in turn can be known only if a specific modified gravity is given a priori.

###  Tsallis entropy

In this subsection we briefly review the concept of nonextensive, or Tsallis entropy [45–47]. As Gibbs pointed out already at 1902, in systems where the partition function diverges, the standard Boltzmann–Gibbs theory is not applicable, and large-scale gravitational systems are known to fall within this class. Tsallis generalized standard thermodynamics (which arises from the hypothesis of weak probabilistic correlations and their connection to ergodicity) to nonextensive one, which can be applied in all cases, and still possessing standard Boltzmann–Gibbs theory as a limit. Hence, the usual Boltzmann–Gibbs additive entropy must be generalized to the nonextensive, i.e non-additive entropy (the entropy of the whole system is not necessarily the sum of the entropies of its sub-systems), which is named Tsallis entropy [45–47, 54, 55]. In cases of spherically symmetric systems that we are interested in this work, it can be written in compact form as [56]:

9 $$\begin{aligned} S_T=\frac{\tilde{\alpha }}{4G} A^{\delta }, \end{aligned}$$

in units where $\hbar =k_B = c = 1$, where $A\propto L^2$ is the area of the system with characteristic length *L*, *G* is the gravitational constant, $\tilde{\alpha }$ is a positive constant with dimensions $[L^{2(1-\delta )}]$ and $\delta$ denotes the non-additivity parameter.^2^ Under the hypothesis of equal probabilities the parameters $\delta$ and $\tilde{\alpha }$ are related to the dimensionality of the system [56] (in particular the important parameter $\delta =d/(d-1)$ for $d>1$), however in the general case they remain independent and free parameters. Obviously, in the case $\delta =1$ and $\tilde{\alpha }=1$, Tsallis entropy becomes the usual Bekenstein–Hawking additive entropy.

### Modified Friedmann equations through nonextensive first law of thermodynamics

In Sect. 2.1 we presented the procedure to extract the Friedmann equations from the first law of thermodynamics. This procedure can be applied in any modified gravity, as long as one knows the black hole entropy relation for this specific modified gravity. Hence, as we mentioned above, although it can be enlightening for the properties of various modified gravities, the thermodynamical approach does not lead to new gravitational modifications since one needs to consider a specific modified gravity a priori.

In the present subsection however, we desire to follow the steps of Sect. 2.1, but instead of the standard additive entropy relation to use the generalized, nonextensive, Tsallis entropy presented in Sect. 2.2 above. Doing so we do obtain modified Friedmann equations, with modification terms that appear for the first time, and which provide the standard Friedmann equations in the case where Tsallis entropy becomes the standard Bekenstein–Hawking one.

We start from the first law of thermodynamics $-dE=TdS$, where $-dE$ is given by (5), *T* by (3), but we will consider that the entropy is given by Tsallis entropy (9). In this case, and recalling that $A=4\pi \tilde{r}_a^2$ we acquire

10 $$\begin{aligned} dS=(4\pi )^\delta \frac{\delta \tilde{\alpha }}{2G}\tilde{r_{a}}^{2\delta -1}\dot{\tilde{r}} _a dt. \end{aligned}$$

Inserting everything in the first law, and calculating $\dot{\tilde{r}}_a$ from (2), we obtain

11 $$\begin{aligned} -\frac{(4\pi )^{2-\delta }G}{\tilde{\alpha }}(\rho _m+p_m)=\delta \frac{\dot{H}-\frac{k}{a^2}}{\left( H^2+\frac{k}{ a^2}\right) ^{\delta -1}}. \end{aligned}$$

Finally, inserting the conservation equation (7) and integrating, for $\delta \ne 2$ we obtain

12 $$\begin{aligned} \frac{2(4\pi )^{2-\delta }G}{3\tilde{\alpha }} \rho _m=\frac{ \delta }{2-\delta } \left( H^2+\frac{k}{a^2}\right) ^{2-\delta }-\frac{\tilde{\Lambda }}{3\tilde{\alpha }}, \end{aligned}$$

where $\tilde{\Lambda }$ is an integration constant. Hence, the use of Tsallis entropy in the first law of thermodynamics, led to two modified Friedmann equations, namely (11) and (12), with modification terms that appear for the first time depending on three parameters out of which two are free.

Let us elaborate the obtained modified Friedmann equations. From now on we focus on the flat case, namely we consider $k=0$, which allows us to extract analytical expressions, however the investigation of the non-flat case is straightforward. We can re-write (11), (12) as

13 $$\begin{aligned} H^2= & {} \frac{8\pi G}{3}\left( \rho _m+\rho _{DE}\right) \end{aligned}$$

14 $$\begin{aligned} \dot{H}= & {} -4\pi G \left( \rho _m+p_m+\rho _{DE}+p_{DE}\right) , \end{aligned}$$

where we have defined the effective dark energy density and pressure as

15 $$\begin{aligned} \rho _{DE}= & {} \frac{3}{8\pi G} \left\{ (4\pi )^{\delta -1}\frac{\tilde{\Lambda }}{3}\right. \nonumber \\&\left. +H^2\left[ 1-\tilde{\alpha }(4\pi )^{ \delta -1}\frac{ \delta }{ 2-\delta } H^{2(1-\delta ) } \right] \right\} , \end{aligned}$$

16 $$\begin{aligned} p_{DE}= & {} -\frac{1}{8\pi G}\left\{ (4\pi )^{\delta -1} \tilde{\Lambda } +2\dot{H}\left[ 1-\tilde{\alpha } (4\pi )^{\delta -1}\delta H^{2(1-\delta )} \right] \right. \nonumber \\&\left. +3H^2\left[ 1-\tilde{\alpha }(4\pi )^{\delta -1}\frac{\delta }{2-\delta }H^{ 2(1-\delta )} \right] \right\} . \end{aligned}$$

We can further simplify the above expressions by redefining $\Lambda \equiv (4\pi )^{\delta -1}\tilde{\Lambda }$ and $\alpha \equiv (4\pi )^{\delta -1}\tilde{\alpha }$, obtaining

17 $$\begin{aligned} \rho _{DE}= & {} \frac{3}{8\pi G} \left\{ \frac{\Lambda }{3}+H^2\left[ 1-\alpha \frac{ \delta }{ 2-\delta } H^{2(1-\delta ) } \right] \right\} , \end{aligned}$$

18 $$\begin{aligned} p_{DE}= & {} -\frac{1}{8\pi G}\left\{ \Lambda +2\dot{H}\left[ 1-\alpha \delta H^{2(1-\delta )} \right] \right. \nonumber \\&\left. +3H^2\left[ 1-\alpha \frac{\delta }{2-\delta }H^{ 2(1-\delta )} \right] \right\} . \end{aligned}$$

Thus, we can define the equation-of-state parameter for the effective dark energy sector as

19 $$\begin{aligned} w_{DE}\equiv \frac{p_{DE}}{\rho _{DE}}=-1- \frac{ 2\dot{H}\left[ 1-\alpha \delta H^{2(1-\delta )} \right] }{\Lambda +3H^2\left[ 1-\frac{\alpha \delta }{2-\delta }H^{2(1-\delta )} \right] } . \end{aligned}$$

In summary, in the constructed modified cosmological scenario, Eqs. (7), (13) and (14) can determine the universe evolution, as long as the matter equation-of-state parameter is known. In particular, inserting (17), (18) into (14), we acquire a differential equation for *H*(*t*) that can be solved similarly to all modified-gravity and dark-energy models.

Finally, as one can see, in the case $\delta =1$ and $\alpha =1$ the generalized Friedmann equations (13), (14) reduce to $\Lambda \hbox {CDM}$ cosmology, namely

20 $$\begin{aligned} H^2= & {} \frac{8\pi G}{3} \rho _m+\frac{\Lambda }{3}\nonumber \\ \dot{H}= & {} -4\pi G(\rho _m+p_m). \end{aligned}$$

We close this subsection by providing for completeness the equations for $\delta =2$. In this special case, integration of (11), instead of (12) results to

21 $$\begin{aligned} \frac{G}{3 \tilde{\alpha }} \rho _m=\ln \left[ H^2+\frac{k}{a^2} \right] -\frac{\tilde{\Lambda }}{6\tilde{\alpha }}. \end{aligned}$$

Hence, in this case the two Friedmann equations (11) and (21), for $k=0$, lead to the definitions

22 $$\begin{aligned} \rho _{DE}= & {} \frac{3}{8\pi G} \left[ \frac{ \Lambda }{3}+H^2-2\alpha \ln H^2 \right] \end{aligned}$$

23 $$\begin{aligned} p_{DE}= & {} -\frac{1}{8\pi G}\left[ \Lambda +3H^2 -6\alpha \ln H^2 +2\dot{H}\left( 1- \frac{2\alpha }{H^2} \right) \right] , \nonumber \\ \end{aligned}$$

and thus

24 $$\begin{aligned} w_{DE}\equiv \frac{p_{DE}}{\rho _{DE}}=-1- \frac{ 2\dot{H}\left( 1- \frac{2\alpha }{H^2} \right) }{\Lambda +3H^2-6\alpha \ln H^2}. \end{aligned}$$

## Cosmological evolution

In this section we proceed to a detailed investigation of the modified cosmological scenarios constructed above. The cosmological equations are the two modified Friedmann equations (13) and (14), along with the conservation equation (7). In the general case of a general matter equation-of-state parameter, $w_m\equiv p_m/\rho _m$, analytical solutions cannot be extracted, and thus one has to solve the above equations numerically. However, we are interested in providing analytical expressions too, and thus in the following we focus to the case of dust matter, namely $w_m=0$.

As usual for convenience we introduce the matter and dark energy density parameters respectively as

25 $$\begin{aligned} \Omega _m= & {} \frac{8\pi G}{3H^2} \rho _m \end{aligned}$$

26 $$\begin{aligned} \Omega _{DE}= & {} \frac{8\pi G}{3H^2} \rho _{DE}. \end{aligned}$$

In the case of dust matter, Eq. (7) gives that $\rho _{m} = \frac{\rho _{m0}}{a^3}$, with $\rho _{m0}$ the value of the matter energy density at present scale factor $a_0=1$ (in the following the subscript “0” marks the present value of a quantity). Therefore, in this case Eq. (25) gives immediately $\Omega _m=\Omega _ {m0} H_{0} ^2/a^3 H^2$. Combining this with the fact that $\Omega _m + \Omega _{DE}=1$ we can easily extract that

27 $$\begin{aligned} H=\frac{\sqrt{\Omega _{m0}} H_{0}}{\sqrt{a^3 (1-\Omega _{DE})}}. \end{aligned}$$

In the following we will use the redshift *z* as the independent variable, defined as $$ 1+z=1/a$$ for $$a_0=1$$. Thus, differentiating (27) we can obtain the useful expression

28 $$\begin{aligned} \dot{H}=-\frac{H^2}{2(1-\Omega _{DE})}[3(1-\Omega _{DE})+(1+z)\Omega '_{DE}], \end{aligned}$$

where a prime denotes derivative with respect to *z*.

Inserting (17) into (26) and using (27) we obtain

29 $$\begin{aligned} \Omega _{DE}(z)= & {} 1-H^{2}_{0}\Omega _{m0}(1+z)^3\nonumber \\&\cdot \left\{ \frac{ (2-\delta )}{\alpha \delta }\left[ H^{2}_{0}\Omega _{m0}(1+z)^3+\frac{\Lambda }{3} \right] \right\} ^{\frac{1}{\delta -2}}. \end{aligned}$$

This expression is the analytical solution for the dark energy density parameter $\Omega _{DE}(z)$, in a flat universe and for dust matter. Applying it at present time, i.e at $z=0$, we acquire

30 $$\begin{aligned} \Lambda =\frac{3\alpha \delta }{2-\delta }H_0^{2(2-\delta )}-3H_0^2\Omega _{m0}, \end{aligned}$$

which provides the relation that relates $\Lambda$, $\delta$ and $\alpha$ with the observationally determined quantities $\Omega _{m0}$ and $H_0$, leaving the scenario with two free parameters. As expected, for $\delta =1$ and $\alpha =1$ all the above relations give those of $\Lambda \hbox {CDM}$ cosmology.

Differentiating (29) we find

31 $$\begin{aligned} \Omega '_{DE}(z)= & {} \left\{ \frac{(2-\delta )}{\alpha \delta }\left[ 1+\frac{\Lambda }{3}\frac{1}{\Omega _{m0}H^{2}_{0}( 1+z)^3}\right] \right\} ^{\frac{3-\delta }{\delta -2}} \nonumber \\&\cdot \frac{1}{\alpha \delta }\left[ \Omega _{m0}H^{2}_{ 0}(1+z)^3\right] ^\frac{1}{\delta -2}\nonumber \\&\cdot \left[ 3(\delta -1)\Omega _{m0}H^{2}_{0}(1+z)^2+(\delta -2)\frac{\Lambda }{1+z}\right] .\nonumber \\ \end{aligned}$$

Hence, we can now calculate the other important observable, namely the dark-energy equation-of-state parameter $w_{DE}$ from (19), eliminating $\dot{H}$ through (28), obtaining

32 $$\begin{aligned}&w_{DE}(z)\nonumber \\&\quad =-1+\frac{\left\{ 3[1-\Omega _{DE}(z)]+(1+z)\Omega _{DE}'(z)\right\} \left\{ 1-\alpha \delta \left[ \frac{ H^{2}_{0}\Omega _{m0}(1+z)^3}{1-\Omega _{DE}(z)}\right] ^{1-\delta }\right\} }{[1-\Omega _{DE} (z)]\left\{ \frac{\Lambda [1-\Omega _{DE}(z)]}{H^{2}_{0}\Omega _{m0}(1+z)^3}+3\left\{ 1-\frac{\alpha \delta }{2-\delta }\left[ \frac{H^{2}_{0}\Omega _{m0}(1+z)^3}{1-\Omega _{DE}(z)}\right] ^{ 1-\delta }\right\} \right\} },\nonumber \\ \end{aligned}$$

where $\Omega _{DE}$ and $\Omega _{DE}'$ are given by (29) and (31) respectively. Lastly, it proves convenient to introduce the deceleration parameter $q\equiv -1-\frac{\dot{H}}{H^2}$, where using (28) is found to be

33 $$\begin{aligned} q(z)=-1+\frac{1}{2[1-\Omega _{DE}(z)]}\{3[1-\Omega _{DE}(z)]+(1+z)\Omega '_{DE}(z)\}. \end{aligned}$$

In summary, considering dust matter and flat geometry we were able to extract analytical solutions for $\Omega _{DE}(z)$ and $w_{DE}(z)$, for the modified, nonextensive cosmological scenarios of the present work. In the following two subsections we will investigate them in two distinct cases, namely when the explicit cosmological constant $\Lambda$ is present and when it is absent.

### Cosmological evolution with $\Lambda \ne 0$

We first examine the case where the explicit cosmological constant $\Lambda$ is present. In this case when $\delta =1$ and $\alpha =1$ we obtain $\Lambda \hbox {CDM}$ cosmology, and thus we are interested in studying the role of the nonextensive parameter $\delta$ on the cosmological evolution.

**Fig. 1:**
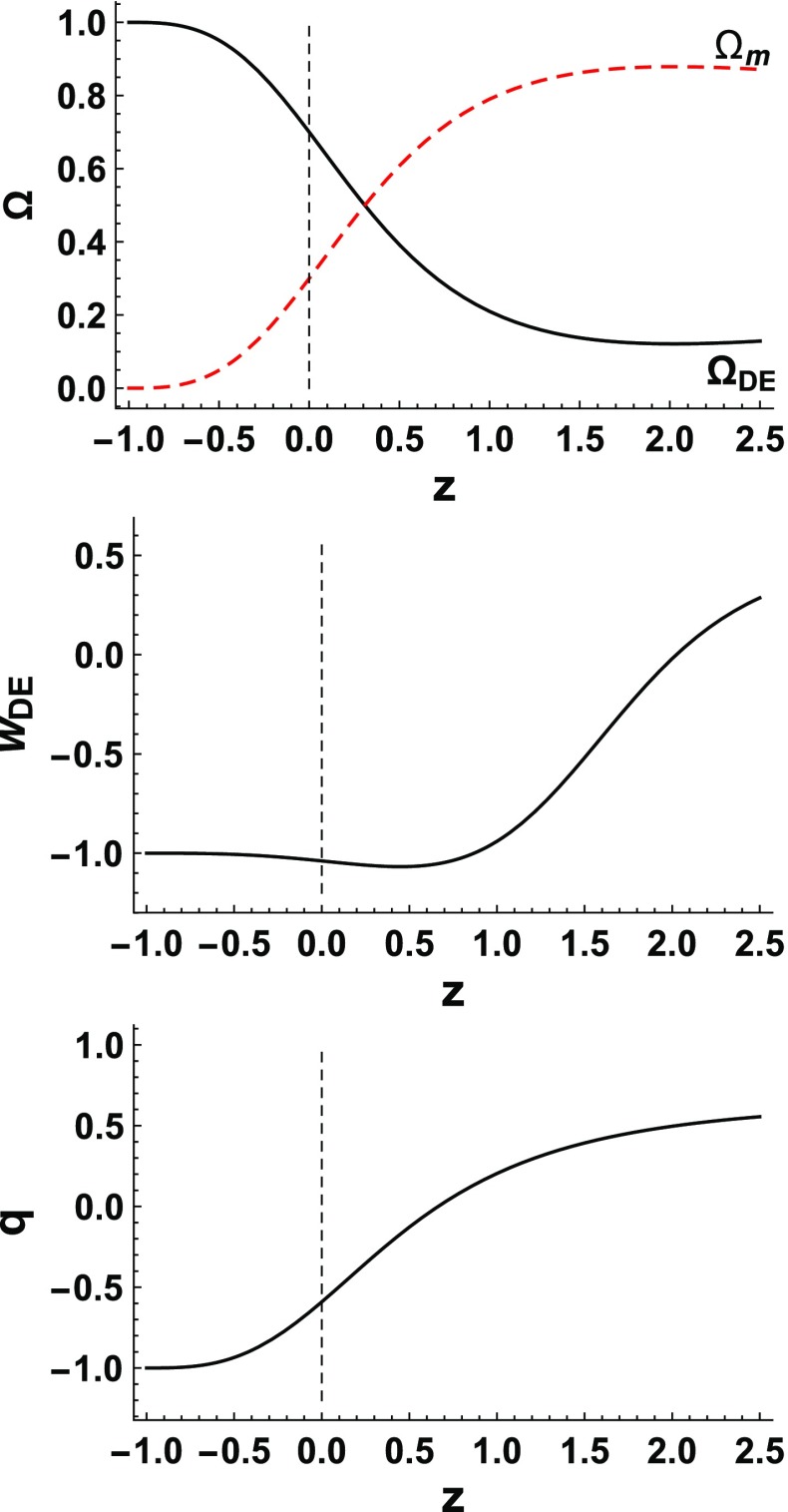
Upper graph: The evolution of the nonextensive dark energy density parameter $\Omega _{DE}$ (black-solid) and of the matter density parameter $\Omega _{m}$ (red-dashed), as a function of the redshift *z*, for $\delta =1.1$ and $\alpha =1$ in units where $H_0=1$. Middle graph: The evolution of the corresponding dark-energy equation-of-state parameter $w_{DE}$. Lower graph: The evolution of the corresponding deceleration parameter *q*. In all graphs we have set the cosmological constant $\Lambda$ from (30) in order to obtain $\Omega _{m}(z=0)=\Omega _{m0}\approx 0.3$ at present, and we have added a vertical dotted line denoting the present time $z=0$

We use relation (30) in order to set the value of $\Lambda$ that corresponds to $\Omega _{m0}\approx 0.3$ in agreement with observations [57]. Moreover, in order to investigate the pure effect of $\delta$, we set $\alpha$ to its standard value, namely $\alpha =1$ (although for $\delta =1$ the parameter $\alpha$ is dimensionless, as we mentioned for $\delta \ne 1$ it acquires dimensions $[L^{2(1-\delta )}]$ and for convenience we use units where $H_0=1$). In the upper graph of Fig. 1 we depict $\Omega _{DE}(z)$ and $\Omega _{m}(z) = 1-\Omega _{DE}(z)$, as given by equation (29), in the case where $\delta =1.1$. In the middle graph we present the corresponding evolution of $w_{DE}(z)$ according to (32). Finally, in the lower graph we present the deceleration parameter *q*(*z*) from (33). We mention that for transparency we have extended the evolution up to the far future, namely up to $z \rightarrow -1$, which corresponds to $t \rightarrow \infty$.

As we observe, we acquire the usual thermal history of the universe, with the sequence of matter and dark energy epochs, with the transition from deceleration to acceleration taking place at $z\approx 0.45$ in agreement with observations. Additionally, in the future the universe tends asymptotically to a complete dark-energy dominated, de-Sitter state. We mention the interesting bahavior that although at intermediate times the dark-energy equation-of-state parameter may experience the phantom-divide crossing and lie in the phantom regime, at asymptotically large times it will always stabilize at the cosmological constant value $-\,1$. Namely, the de-Sitter solution is a stable late-time attractor, which is a significant advantage (this can be easily showed taking the limit $z \rightarrow -1$ in (29),(31) and (32), which gives $\Omega _{DE}\rightarrow 1$, $\Omega '_{DE}\rightarrow 0$, and $w_{DE}\rightarrow -1$, respectively).

**Fig. 2:**
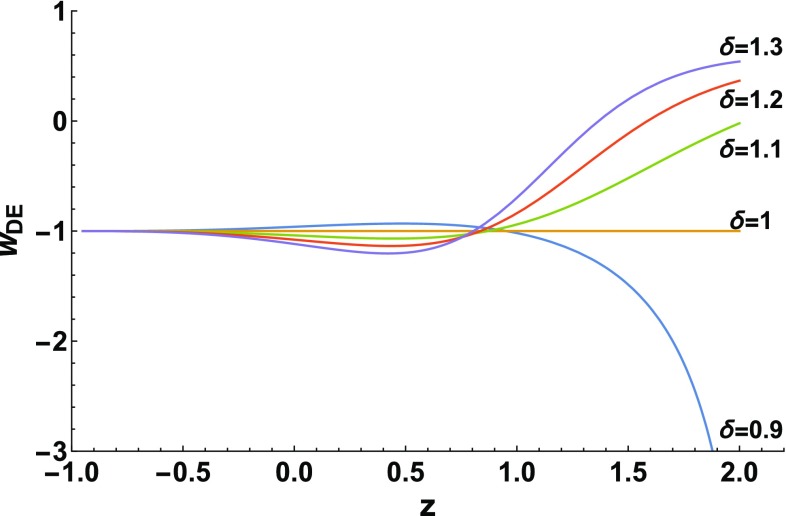
The evolution of the dark-energy equation-of-state parameter $w_{DE}$ as a function of the redshift *z*, for $\alpha =1$ in units where $H_0=1$, and various values of the nonextensive parameter $\delta$. For each value of $\delta$ we choose $\Lambda$ according to (30) in order to obtain $\Omega _{m}(z=0)=\Omega _{m0}\approx 0.3$ at present, and acquire an evolution of $\Omega _{DE}$ and $\Omega _m$ similar to the upper graph of Fig. 1

Let us now examine in detail the role of $\delta$ in the evolution, and in particular on $w_{DE}$. In Fig. 2 we depict $w_{DE}(z)$ for $\alpha =1$ and for various values of $\delta$, including the value $\delta =1$ that reproduces $\Lambda \hbox {CDM}$ cosmology. For each value of $\delta$ we choose $\Lambda$ according to (30) in order to obtain $\Omega _{m}(z=0)=\Omega _{m0}\approx 0.3$ at present, and obtain an evolution of $\Omega _{DE}(z)$ and $\Omega _m(z)$ similar to the upper graph of Fig. 1. In this way we can examine the pure effect of $\delta$. Firstly, as we mentioned, for $\delta =1$ we obtain $w_{DE}=-1=const.$, namely $\Lambda \hbox {CDM}$ cosmology. For increasing $\delta >1$, at earlier redshifts $w_{DE}$ acquires larger values, while on the contrary in the recent past, i.e at $0\le z\lesssim 0.8$, $w_{DE}$ acquires algebraically smaller values, which is also true for its present value $w_{DE0}$. In all cases the universe experiences the phantom-divide crossing, and in the far future it results from below in a de-Sitter phase with $w_{DE}$ being $-\,1$. On the other hand, for decreasing $\delta <1$ the behavior of $w_{DE}(z)$ is the opposite, namely it initially lies in the phantom regime, it then crosses the $-\,1$-divide from below to above being quintessence-like at present, and finally it asymptotically tends to $-\,1$ from above.

In summary, we can see that the nonextensive parameter $\delta$, that lies in the core of the modified cosmology obtained in this work, plays an important role in giving to dark energy a dynamical nature and bringing about a correction to $\Lambda \hbox {CDM}$ cosmology. We mention that in all the above examples we kept the parameter $\alpha$ fixed, in order to maintain the one-parameter character of the scenario. Clearly, letting $\alpha$ vary too, increases the capabilities of the model and the obtained cosmological behaviors.

### Cosmological evolution with $\Lambda =0$

In the previous subsection we investigated the scenario of modified Friedmann equations through nonextensive thermodynamics, in the case where the cosmological constant is explicitly present. Thus, we studied models that possess $\Lambda \hbox {CDM}$ cosmology as a subcase, and in which the nonextensive parameter $\delta$ and its induced novel terms lead to corrections to $\Lambda \hbox {CDM}$ paradigm.

In the present subsection we are interested in studying a more radical application of the scenario at hand, namely to consider that an explicit cosmological constant is not present and let the model parameters $\delta$ and $\alpha$ to mimic its behavior and produce a cosmology in agreement with observations.

In the case $\Lambda =0$, relations (17), (18) become

34 $$\begin{aligned} \rho _{DE}= & {} \frac{3}{8\pi G}H^2\left[ 1-\alpha \frac{ \delta }{ 2-\delta } H^{2(1-\delta ) } \right] \end{aligned}$$

35 $$\begin{aligned} p_{DE}= & {} -\frac{1}{8\pi G}\left\{ 3H^2\left[ 1-\alpha \frac{\delta }{2-\delta }H^{ 2(1-\delta )} \right] \right. \nonumber \\&\left. +2\dot{H}\left[ 1-\alpha \delta H^{2(1-\delta )} \right] \right\} , \end{aligned}$$

while (29) reads

36 $$\begin{aligned} \Omega _{DE}(z)=1-\left\{ \frac{(2-\delta )}{\alpha \delta } \left[ \Omega _{m0}H_0^2 (1+z)^3\right] ^{\delta -1} \right\} ^{\frac{1}{\delta -2}}. \end{aligned}$$

However, the important simplification comes from expression (30), that relates $\Lambda$ and $\alpha$ with the observationally determined quantities $\Omega _{m0}$ and $H_0$. In particular, setting $\Lambda =0$ leads to the determination of parameter $\alpha$ in terms of $\Omega _{m0}$ and $H_0$, namely

37 $$\begin{aligned} \alpha =\frac{(2-\delta )}{\delta }\Omega _{m0}H^{2(\delta -1)}_{0}, \end{aligned}$$

leaving $\delta$ as the only free model parameter. Note that since $\tilde{\alpha }>0$ in (9), i.e $\alpha >0$, from (37) we deduce that the present scenario is realized for $\delta <2$. Thus, inserting (37) into (36) leads to the simplified expression

38 $$\begin{aligned} \Omega _{DE}(z)=1-\Omega _{m0}(1+z)^{\frac{3(\delta -1)}{\delta -2}}. \end{aligned}$$

Finally, inserting (37) and (38) into (32) and (33) gives respectively

39 $$\begin{aligned} w_{DE}(z)=\frac{(\delta -1)}{(2-\delta )} \left[ 1-\Omega _{m0}(1+z)^\frac{3(\delta -1)}{(\delta - 2)} \right] ^{-1}, \end{aligned}$$

and

40 $$\begin{aligned} q(z)=\frac{2\delta -1}{2(2-\delta )}. \end{aligned}$$

We stress here that in this case exact $\Lambda \hbox {CDM}$ cosmology cannot be obtained for any parameter values, and thus one should suitably choose $\delta$ in order to acquire agreement with observations. Note that in the standard extensive choice $\delta =1$ we obtain a trivial universe with $\Omega _{DE}(z)=1-\Omega _{m0}=const.$ and $w_{DE}(z)=0$.

From the analytical expression (38) we can see that we acquire the thermal history of the universe, with the sequence of matter and dark energy epochs and the onset of late-time acceleration. Furthermore, in the future ($z \rightarrow -1$) the universe tends asymptotically to the complete dark-energy domination. Additionally, as can be seen from expression (39), the asymptotic value of $w_{DE}$ in the far future is not necessarily the cosmological constant value $-1$. In particular, we deduce that for $1\le \delta <2$ $w_{DE}\rightarrow 0$ as $z \rightarrow -1$, while for $\delta <1$ $w_{DE}\rightarrow (\delta -1)/(2-\delta )$ as $z \rightarrow -1$. Hence, the case $\delta <1$ is the one that exhibits more interesting behavior in agreement with observations, and we observe that for decreasing $\delta$ the $w_{DE}(z)$ tends to lower values.

We close this subsection mentioning that according to the above analysis the cosmological behavior is very efficient for low redshifts and up to the far future, despite the fact that an explicit cosmological constant is absent. However, as can be seen from (36), for high redshifts the behavior of $\Omega _{DE}(z)$ is not satisfactory, since as it is this expression leads to either early-time dark energy or to the unphysical result that $\Omega _{DE}(z)$ becomes negative. In order to eliminate this behavior and obtain a universe evolution in agreement with observations at all redshifts one needs to include the radiation sector too, which indeed can regulate the early-time behavior. This is performed in the next subsection.

### Cosmological evolution including radiation

In this subsection for completeness we extend the scenario of modified cosmology through nonextensive horizon thermodynamics, in the case where the radiation fluid is also present. First of all, in the case where extra fluids are considered in the universe content, the thermodynamical procedure of Sect. 2 is applicable in exactly the same way, with the only straightforward addition being that in Eq. (5) one should add the energy densities and pressures of all universe fluids [31–34, 42, 53]. Hence, if we allow for a radiation fluid, with energy density $\rho _r$ and pressure $p_r$, and repeat the analysis of Sect. 2.3, the Friedmann equations (13), (14) become

41 $$\begin{aligned} H^2= & {} \frac{8\pi G}{3}\left( \rho _m+\rho _r+\rho _{DE}\right) \end{aligned}$$

42 $$\begin{aligned} \dot{H}= & {} -4\pi G \left( \rho _m+p_m+\rho _r+p_r+\rho _{DE}+p_{DE}\right) , \end{aligned}$$

with $\rho _{DE}$, $p_{DE}$ still given by (17), (18), and $w_{DE}$ by (19).

We proceed by introducing the radiation density parameter as

43 $$\begin{aligned} \Omega _r\equiv \frac{8\pi G}{3H^2}\rho _r, \end{aligned}$$

and thus the first Friedmann equation becomes $\Omega _r+ \Omega _m+\Omega _{DE}=1$. Similarly to the analysis of Sect.  3, in order to extract analytical expressions we consider that the matter fluid is dust, namely $w_m=0$. In the case where radiation is present we still have that $\Omega _m=\Omega _ {m0} H_{0} ^2/a^3 H^2$, however (27) now extends to

44 $$\begin{aligned} H=\frac{\sqrt{\Omega _{m0}} H_{0}}{\sqrt{a^3 (1-\Omega _{DE}-\Omega _{r})}}, \end{aligned}$$

while (28) reads

45 $$\begin{aligned} \dot{H}=-\frac{H^2}{2}\left[ \frac{3\Omega _{m0}+4\Omega _{r0}(1+z)}{\Omega _{m0} +\Omega _{ r0}(1+z)}+\frac{(1+z)\Omega _{DE}'}{(1-\Omega _{DE})}\right] , \end{aligned}$$

since for dust matter we have

46 $$\begin{aligned} \Omega _{r}(z)=\frac{\Omega _{r0}(1+z)^{4}[1-\Omega _{DE}(z)]}{\Omega _{m0} (1+z)^{3}+\Omega _{ r0 } (1+z)^{4}}. \end{aligned}$$

#### Cosmological evolution with $\Lambda \ne 0$

Let us first investigate the case where $\Lambda \ne 0$. Inserting (17) into (26) and using (44) we find that (29) extends to

47 $$\begin{aligned} \Omega _{DE}(z)= & {} 1-H^{2}_{0}\left[ \Omega _{m0}(1+z)^3+\Omega _{r0}(1+z)^4\right] \nonumber \\&\cdot \left\{ \frac{ (2-\delta )}{\alpha \delta }\left[ H^{2}_{0}\left[ \Omega _{m0}(1+z)^3+\Omega _{r0}(1+z)^4\right] +\frac{ \Lambda }{ 3} \right] \right\} ^{\frac{1}{\delta -2}}.\nonumber \\ \end{aligned}$$

This expression is the analytical solution for the dark energy density parameter $\Omega _{DE}(z)$, in a flat universe and for dust matter, in the case where radiation is present. Applying it at present time, i.e at $z=0$, we acquire

48 $$\begin{aligned} \Lambda =\frac{3\alpha \delta }{2-\delta }H^{2(2-\delta )}_{0}-3H^{2}_{0}\left( \Omega _{m0}+\Omega _{r0}\right) , \end{aligned}$$

which provides the relation that relates $\Lambda$, $\delta$ and $\alpha$ with the observationally determined quantities $\Omega _{m0}$, $\Omega _{r0}$ and $H_0$, leaving the scenario with two free parameters. As expected, for $\delta =1$ and $\alpha =1$ all the above relations give those of $\Lambda \hbox {CDM}$ cosmology with radiation sector present.

Differentiating (47) we find

49 $$\begin{aligned} \Omega '_{DE}(z)=\mathcal {A}(z)\mathcal {B}^{\frac{1}{\delta -2}}(z)\left[ \frac{1}{\alpha \delta }\mathcal {B}^{-1}(z)\mathcal {C} (z)-1\right] , \end{aligned}$$

where $\mathcal {A}(z)=H^{2}_{0}\left[ 3\Omega _{m0}(1+z)^2+4\Omega _{r0}(1+z)^3\right]$, $\mathcal {B}(z)=\frac{2-\delta }{\alpha \delta }\left[ H^{2}_{0}\left[ \Omega _{m0}(1+z)^3+\Omega _{r0}( 1+z)^4\right] +\frac{\Lambda }{3}\right]$ and $\mathcal {C}(z)=H^{2}_{0}\left[ \Omega _{m0}(1+z)^3+\Omega _{r0}(1+z)^4\right]$. Hence, $w_{DE}(z)$ is calculated from (19), but now eliminating $\dot{H}$ through (45), obtaining

50 $$\begin{aligned}&w_{DE}(z)\nonumber \\&\quad =-1+\frac{\left[ \frac{3\Omega _{m0}+4\Omega _{r0}(1+z)}{\Omega _{m0}+\Omega _{ r0}(1+z)}(1-\Omega _{ DE})+(1+z)\Omega _{DE}'\right] \left\{ 1-\alpha \delta \left[ \frac{H^{2}_{0}\left[ \Omega _{m0}(1+z)^3+\Omega _{r0}(1+z)^4\right] }{(1-\Omega _{DE})} \right] ^{1-\delta }\right\} }{(1-\Omega _{DE} )\left\{ \frac{\Lambda (1-\Omega _{DE})}{H^{2}_{0}\left[ \Omega _{m0}(1+z)^3+\Omega _{r0}(1+z)^4\right] }+3\left\{ 1-\frac{\alpha \delta }{2-\delta }\left\{ \frac{H^{2}_{0}\left[ \Omega _{m0}(1+z)^3+\Omega _{r0}(1+z)^4\right] }{(1-\Omega _{DE})}\right\} ^{1-\delta }\right\} \right\} }, \end{aligned}$$

where $\Omega _{DE}$ and $\Omega _{DE}'$ are given by (47) and (49) respectively. Lastly, the deceleration parameter $q\equiv -1-\frac{\dot{H}}{H^2}$, using (45) is found to be

51 $$\begin{aligned} q(z)=-1+\frac{1}{2}\left[ \frac{3\Omega _{m0}+4\Omega _{r0}(1+z)}{\Omega _{m0} +\Omega _{ r0}(1+z)}+\frac{(1+z)\Omega _{DE}'}{(1-\Omega _{DE})}\right] . \end{aligned}$$

In summary, in the case where radiation is present, we were able to extract analytical solutions for $\Omega _{DE}(z)$ and $w_{DE}(z)$, for the modified, nonextensive cosmological scenarios of the present work.

#### Cosmological evolution with $\Lambda = 0$

Let us now focus on the interesting case where the explicit cosmological constant is absent, namely when $\Lambda =0$. This scenario was analyzed in Sect. 3.2 above in the absence of radiation, however we now study it in the full case where radiation is included. For $\Lambda =0$, relation (47) becomes

52 $$\begin{aligned} \Omega _{DE}(z)=1-\left\{ \frac{\left[ \Omega _{m0}(1+z)^{3}+\Omega _{r0}(1+z)^{4}\right] ^{ \delta -1}}{\Omega _{m0}+\Omega _{r0}}\right\} ^{\frac{1}{\delta -2}}, \end{aligned}$$

relation (48) becomes

53 $$\begin{aligned} \alpha =\frac{2-\delta }{\delta }H^{2(\delta -1)}_{0}\left[ \Omega _{m0}+\Omega _{r0}\right] , \end{aligned}$$

and thus positivity of $\alpha$ implies that $\delta <2$, relation (49) becomes

54 $$\begin{aligned} \Omega _{DE}'(z)= & {} -\frac{\delta -1}{\delta -2}\left\{ \frac{\left[ \Omega _{m0}(1+z)^3+\Omega _{r0}(1+z)^4\right] }{\left[ \Omega _{m0} +\Omega _{r0}\right] }\right\} ^{\frac{1}{\delta -2}}\nonumber \\&\cdot \left[ 3\Omega _{m0}(1+z) ^2+4\Omega _{r0}(1+z)^3\right] , \end{aligned}$$

relation (50) becomes

55 $$\begin{aligned} w_{DE}(z)= & {} \frac{3(1-\delta )\Omega _{m0}+(2-3\delta )(1+z)\Omega _{r0} }{3(\delta -2) [\Omega _{m0}+(1+z)\Omega _{r0}]} \nonumber \\&+ \frac{(\delta -1) [3\Omega _{m0}+4(1+z)\Omega _{r0}]}{3(\delta -2) [\Omega _{m0}+(1+z)\Omega _{r0}]- 3(\delta -2) (1+z)^{\frac{3(1-\delta )}{\delta -2}}(\Omega _{m0}+\Omega _{r0})^{\frac{1}{\delta -2}}[\Omega _ {m0}+(1+z)\Omega _{r0}]^{\frac{1}{2-\delta }}}, \end{aligned}$$

while relation (51) becomes

56 $$\begin{aligned} q= & {} \frac{\left[ \Omega _{m0}+2\Omega _{r0}(1+z)\right] }{2\left[ \Omega _{m0}+\Omega _{ r0} (1+z)\right] } \nonumber \\&-\frac{\delta -1}{2(\delta -2)}\left\{ \frac{\left[ 3\Omega _{m0}(1+z)^3+4\Omega _{r0}(1+z)^4\right] }{\left[ \Omega _{m0} (1+z)^3+\Omega _{r0}(1+z)^4\right] }\right\} . \end{aligned}$$

We mention that relations (52)–(56) are the extensions of (36)–(40) in the presence of radiation.

**Fig. 3:**
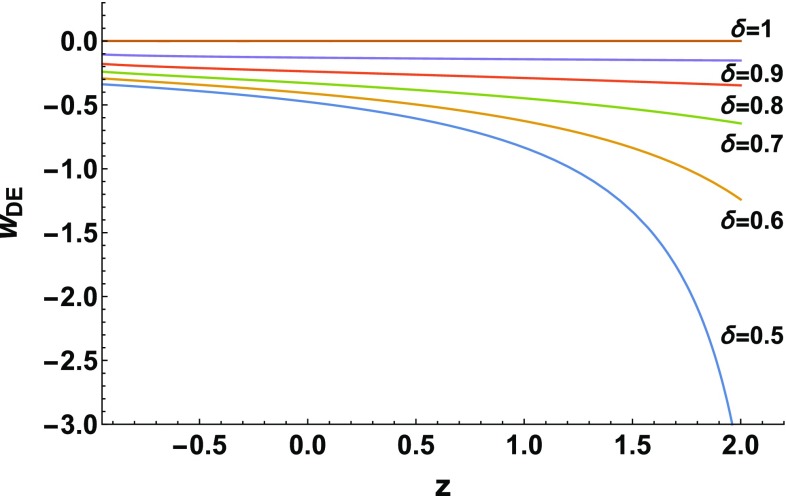
The evolution of the equation-of-state parameter $w_{DE}$ as a function of the redshift *z*, for $\Lambda =0$ and for various values of the nonextensive parameter $\delta$, in the case where radiation is present. For each value of $\delta$ we choose $\alpha$ according to (53) in order to obtain $\Omega _{m}(z=0)=\Omega _{m0}=0.3$ and $\Omega _{r}(z=0)=\Omega _{r0}=0.000092$ at present [57], and acquire the expected thermal history of the universe

Let us examine this scenario in more detail, and in particular study the effect of $\delta$ on the cosmological evolution. In Fig. 3 we present $w_{DE}(z)$ for various choices of $\delta$, extending the evolution up to the far future. In all cases the parameter $\alpha$ is set according to (53) in order to obtain $\Omega _{m}(z=0)=\Omega _{m0}=0.3$ and $\Omega _{r}(z=0)=\Omega _{r0}=0.000092$ [57], and the expected thermal history of the universe. As we observe, for decreasing $\delta$ the $w_{DE}(z)$ tends to lower values. Moreover, although the asymptotic value of $\Omega _{DE}(z)$ as $z \rightarrow -1$ is 1, as can be seen immediately from (52), namely the universe tends to the complete dark-energy domination, the asymptotic value of $w_{DE}$ is not the cosmological constant value $-1$, i.e the universe does not result in a de Sitter space. In particular, from (55) we can see that for $1\le \delta <2$, $w_{DE}\rightarrow 0$ as $z \rightarrow -1$, while for $\delta <1$, $w_{DE}\rightarrow (\delta -1)/(2-\delta )$ as $z \rightarrow -1$. These asymptotic values are the same with the ones in the absent of radiation mentioned in Sect. 3.2, which was expected since at late times the effect of radiation is negligible.

In summary, the scenario of modified cosmology through nonextensive thermodynamics, even in the case where an explicit cosmological constant is absent, is efficient in describing the cosmological behavior of the universe. In order to present this behavior more transparently we confront the scenario with Supernovae type Ia (SN Ia) data. In these observational sets the apparent luminosity *l*(*z*), or equivalently the apparent magnitude *m*(*z*), are measured as functions of the redshift, and are related to the luminosity distance as

57 $$\begin{aligned} 2.5 \log \left[ \frac{L}{l(z)}\right] = \mu \equiv m(z) - M = 5 \log \left[ \frac{d_L(z)_{\text {obs}}}{Mpc}\right] + 25, \end{aligned}$$

where *M* and *L* are the absolute magnitude and luminosity respectively. Additionally, for any theoretical model one can calculate the predicted dimensionless luminosity distance $d_{L}(z)_\text {th}$ using the predicted evolution of the Hubble function as

58 $$\begin{aligned} d_{L}\left( z\right) _\text {th}\equiv \left( 1+z\right) \int ^{z}_{0}\frac{dz'}{H\left( z'\right) }~. \end{aligned}$$

**Fig. 4:**
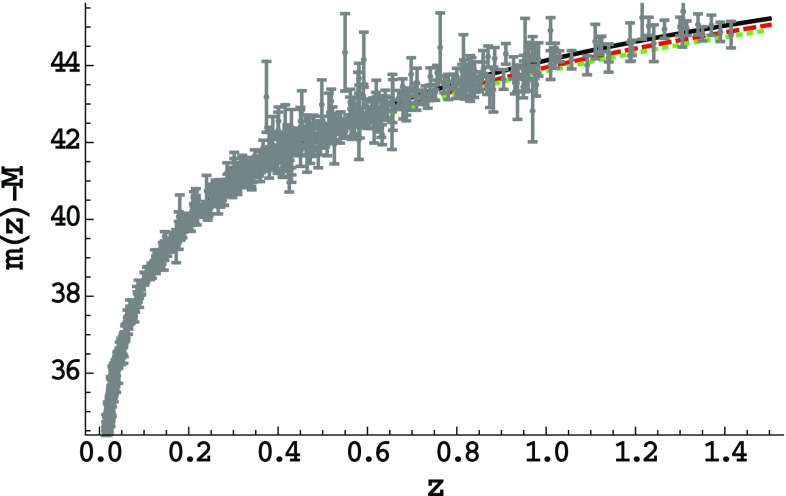
The theoretically predicted apparent minus absolute magnitude as a function of the redshift, for the scenario of modified cosmology through nonextensive thermodynamics, for $\Lambda =0$, in the case where radiation is present, for $\delta =0.5$ (red-dashed) and $\delta =0.6$ (green-dotted). The observational points correspond to the 580 SN Ia data points from [58], and for completeness and comparison we depict the prediction of $\Lambda \hbox {CDM}$ cosmology with the black-solid curve

In the scenario at hand, *H*(*z*) can be immediately calculated analytically from (44), knowing (46) and (47). In Fig. 4 we depict the theoretically predicted apparent minus absolute magnitude as a function of *z*, for two $\delta$ choices, as well as the prediction of $\Lambda \hbox {CDM}$ cosmology, on top of the 580 SN Ia observational data points from [58]. As we can see the agreement with the SN Ia data is excellent. The detailed comparison with observations, namely the joint analysis using data from SN Ia, Baryon Acoustic Oscillation (BAO), Cosmic Microwave Background (CMB), and direct Hubble parameter observations, lies beyond the scope of the present work and it is left for a future project.

We close this subsection mentioning that the present scenario is very efficient in mimicking the cosmological constant, despite the fact that in this case the exact $\Lambda \hbox {CDM}$ cosmology cannot be obtained for any parameter values. In particular, choosing the nonextensive parameter $\delta$ suitably (namely $\delta \sim 0.5-0.6$) we acquire agreement with observations. This is a significant result that shows the capabilities of the modified cosmology through nonextensive thermodynamics.

## Conclusions

In this work we constructed a modified cosmological scenario through the application of the first law of thermodynamics, but using the generalized, nonextensive Tsallis entropy instead of the usual Bekenstein–Hawking one. In particular, there is a well-studied procedure in the literature, which works for a variety of modified gravities, where one can apply the first law of thermodynamics in the universe horizon and extract the Friedmann equations. The crucial part in this procedure is the use of the modified entropy relation of the specific modified gravity, which is known only after this modified gravity is given, and thus in this sense it cannot provide new gravitational modifications. However, if we apply this approach using the nonextensive, Tsallis entropy, which is the consistent concept that should be used in non-additive gravitational systems such us the whole universe, then we result to modified cosmological equations that possess the usual ones as a particular limit, but which in the general case contain extra terms that appear for the first time.

The new terms that appear in the modified Friedmann equations are quantified by the nonextensive parameter $\delta$ and constitute an effective dark energy sector. In the case where Tsallis entropy becomes the usual Bekenstein–Hawking entropy, namely when $\delta =1$, the effective dark energy coincides with the cosmological constant and $\Lambda \hbox {CDM}$ cosmology is restored. However, in the general case the scenario of modified cosmology at hand presents very interesting cosmological behavior.

When the matter sector is dust, we were able to extract analytical expressions for the dark energy density and equation-of-state parameters, and we extended these solutions in the case where radiation is present too. These solutions show that the universe exhibits the usual thermal history, with the sequence of matter and dark-energy eras and the onset of acceleration at around $z\approx 0.5$ in agreement with observations. In the case where an explicit cosmological constant is present, according to the value of $\delta$ the dark-energy equation-of-state parameter exhibits a very interesting behavior and it can be quintessence-like, phantom-like, or experience the phantom-divide crossing during the evolution, before it asymptotically stabilizes in the cosmological constant value $-\,1$ in the far future.

An interesting sub-case of the scenario of modified cosmology through nonextensive thermodynamics is when we set the explicit cosmological constant to zero, since in this case the universe evolution is driven solely by the news terms. Extracting analytical solutions for the dark energy density and equation-of-state parameters we showed that indeed the new terms can very efficiently mimic $\Lambda \hbox {CDM}$ cosmology, although $\Lambda$ is absent, with the successive sequence of matter and dark energy epochs, before the universe results in complete dark-energy domination in the far future. Moreover, confronting the model with SN Ia data we saw that the agreement is excellent.

In summary, modified cosmology through nonextensive thermodynamics is very efficient in describing the universe evolution, and thus it can be a candidate for the description of nature. In the present work we derived the cosmological equations by applying the well-known thermodynamics procedure to the universe horizon. It would be interesting to investigate whether these equations can arise from a nonextensive action too. Such a study is left for a future project.

## Footnotes

1 Note that although this will certainly be the situation at late times, when the universe fluid and the horizon will have interacted for a long time, it is not assured that it will be the case at early or intermediate times. However, in order to avoid applying non-equilibrium thermodynamics, which leads to mathematical complexity, the assumption of equilibrium is widely used [31–34, 42, 53]. Thus, we will follow this assumption and we will have in mind that our results hold only at late times of the universe evolution.

2 In Ref. [56] the nonextensive entropy relation is written as $S_T=\gamma A^\delta$, however we prefer to write is as in (9) for convenience.
